# Development of an Effective Tumor-Targeted Contrast Agent for Magnetic Resonance Imaging Based on Mn/H-Ferritin Nanocomplexes

**DOI:** 10.1021/acsabm.1c00724

**Published:** 2021-10-19

**Authors:** Chiara Tullio, Lucia Salvioni, Michela Bellini, Anna Degrassi, Luisa Fiandra, Massimiliano D’Arienzo, Stefania Garbujo, Rany Rotem, Filippo Testa, Davide Prosperi, Miriam Colombo

**Affiliations:** † NanoBioLab, Department of Biotechnology and Bioscience, University of Milano-Bicocca, Piazza della Scienza 2, 20126 Milano, Italy; ‡ Preclinical Development, Efficacy and Safety, Accelera S.R.L.—NMS Group S.p.A., viale Pasteur 10, 20014 Nerviano, MI, Italy; § Department of Materials Science, University of Milano-Bicocca, Via Roberto Cozzi 55, 20125 Milano, Italy

**Keywords:** MRI, manganese, H-ferritin, tumor targeting, contrast agent, diagnostic imaging

## Abstract

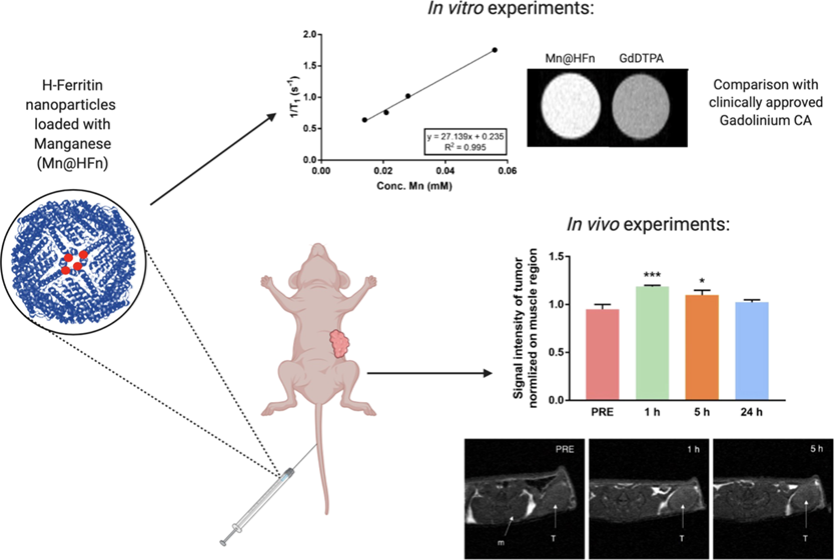

Magnetic resonance imaging (MRI) is one of the most sophisticated diagnostic tools that is routinely used in clinical practice. Contrast agents (CAs) are commonly exploited to afford much clearer images of detectable organs and to reduce the risk of misdiagnosis caused by limited MRI sensitivity. Currently, only a few gadolinium-based CAs are approved for clinical use. Concerns about their toxicity remain, and their administration is approved only under strict controls. Here, we report the synthesis and validation of a manganese-based CA, namely, Mn@HFn-RT. Manganese is an endogenous paramagnetic metal able to produce a positive contrast like gadolinium, but it is thought to result in less toxicity for the human body. Mn ions were efficiently loaded inside the shell of a recombinant H-ferritin (HFn), which is selectively recognized by the majority of human cancer cells through their transferrin receptor 1. Mn@HFn-RT was characterized, showing excellent colloidal stability, superior relaxivity, and a good safety profile. In vitro experiments confirmed the ability of Mn@HFn-RT to efficiently and selectively target breast cancer cells. In vivo, Mn@HFn-RT allowed the direct detection of tumors by positive contrast enhancement in a breast cancer murine model, using very low metal dosages and exhibiting rapid clearance after diagnosis. Hence, Mn@HFn-RT is proposed as a promising CA candidate to be developed for MRI.

## Introduction

The early detection of cancer is a huge and important challenge in clinical settings. The timing of diagnosis is essential for increasing the chance of efficiently treating and finally eradicating tumor formations.^1^ Magnetic resonance imaging (MRI) is a technique that is able to capture the images of inner body regions by exploiting the principle of nuclear magnetic resonance (NMR), which is based on differences in the longitudinal (*T*_1_) and transverse (*T*_2_) relaxation time of the hydrogen spin of water molecules. MRI is widely used in clinical settings due to its noninvasiveness and the fact that it does not involve the use of radioactive substances. To enhance the contrast within different tissues and facilitate the detection of abnormal regions, it is possible to inject contrast agents (CAs), particularly metal-based compounds capable of decreasing the relaxation times of *T*_1_ and *T*_2_ of the interacting water molecules present in the body. The results are brighter (*T*_1_) or darker (*T*_2_) weighted images.^2^

At the present time, the metal of choice for MRI CAs is gadolinium (Gd), a paramagnetic element that is able to improve the resolution of *T*_1_-weighted images. A Gd(III)-based formulation requires ion complexation by means of specific chelating molecules to allow the element’s rapid clearance and avoid long-term toxic effects.^3^ Nevertheless, its widespread use in clinical settings is controversial and strictly controlled. Some research has reported a correlation between Gd chelates and nephrogenic systemic fibrosis in people with renal disease, establishing contraindications for patients with renal issues.^4,5^ Moreover, in 2017, the European Medicines Agency withdrew from the market some linear Gd-based CAs (e.g., Magnevist, Omniscan, and Optimark) and restricted the use of a few others because evidence of Gd has been found in the brains of patients, pointing to certain potential risks.^6,7^

Manganese (Mn) has been proposed as a less toxic alternative. Indeed, its presence in the body is already essential for human health because of its role as a fundamental proactive cofactor for several enzymes.^8^ Mn(II) possesses five unpaired electrons and thus is considered a high-spin paramagnetic metal. Despite these advantages, only one injectable Mn(II)-based MRI CA has been approved for clinical use to date (Teslascan),^9,10^ and it was withdrawn from the marketplace (in 2003 in the United States and 2010 in the EU) after a few years of use due to poor clinical performance and toxicity concerns. Many compounds are currently under investigation, including new Mn chelates,^11^ MnO nanoparticles (NPs),^12^ and Mn-based liposome CAs.^13^

A promising strategy, already investigated with Gd,^14−16^ is the encapsulation of paramagnetic ions inside a protecting shell of safe biological nanocarriers such as apoferritin.^17,18^ This spherical protein has a diameter of 12 nm and an inner cavity of 8 nm and is present in the majority of living species; it has a double function of storing iron and providing a cellular defense against free radicals.^19^ Apoferritin is composed of 24 subunits, each of which contains a mixture of two homologues, heavy (H) chain and light (L) chain, present in different ratios depending on the various species and tissues in which it resides.^20^ The entrapment of Fe as ferrihydrite is possible due to ferroxidase sites present in the H chain and a negatively charged inner surface that is rich in acidic amino acids.^21^ The presence of these regions rich in chelating groups in the inner cavity of apoferritin suggested that positively charged metal ions could be favorably accommodated inside.

It has been observed that the ligand preferences and binding sites of Fe and Mn are very similar for many enzymes.^22^ In particular, Mn can bind to a ferroxidase center in native apoferritin, which influences the efficiency of the ferroxidation reaction, while Fe loading in the presence of Mn originates a core containing both metal ions.^23,24^ All findings suggest that apoferritin has a natural inclination also to encapsulate Mn. The structure of the protein may also increase the efficiency of Mn as a CA. In fact, water can exchange between the core and the outside of apoferritin through narrow intermonomer channels, and the inner surface of the protein may enhance relaxivity via catalytic exchange of water protons, as has already been demonstrated using Gd.^25^

Mn was encapsulated in apoferritin for the first time in 1995, producing an inner core of Mn(III) oxyhydroxide.^26^ Afterward, a number of different studies were conducted to promote the encapsulation of Mn(II), demonstrating its potentiality as a CA through a detailed characterization of the relevant compounds.^27,28^ Thus far, in vivo studies using Mn-loaded apoferritin NPs have been focused on clearance organs, mainly targeting hepatocarcinoma lesions in a mouse model.^29^

In the present work, our goal was to fully exploit the potential of H-ferritin (HFn)—a recombinant variant of human apoferritin consisting of 24 self-assembled heavy-chain subunits—to produce a CA able to detect various kinds of tumor lesions and thus able to target solid tumors disseminated in tissues other than the liver. In fact, it has been demonstrated that transferrin receptor 1 (TfR1), a receptor recognizing human transferrin and HFn subunits, is overexpressed in 98% of human tumor cells.^30^ HFn nanoparticles can discriminate between malignant and normal cells and are preferentially internalized by cancer cells.^31^ Therefore, to investigate the potential of HFn as a safe, biocompatible, and tumor-targeted alternative CA chelating system, we have produced a recombinant HFn stably loaded with Mn ions. We have explored various strategies and selected the most promising CA, which was then tested in vitro and in a breast cancer mouse model. The enhanced brightness in the tumor areas allowed the detection of abnormal masses.

## Materials and Methods

### Cell Lines

HCC1954 and HeLa, human tumor cell lines from the mammary gland and cervix, respectively, were used as a TfR1-positive model of cancer cells, while murine fibroblasts NIH-3T3 were selected as a healthy cell line. HCC1954 cells were cultured in Dulbecco’s modified Eagle’s medium (DMEM) supplemented with 10% fetal bovine serum (FBS), 2 mM of l-glutamine, penicillin (50 IU/mL), and streptomycin (50 mg/mL), while the HeLa and NIH-3T3 media were composed of Roswell Park Memorial Institute Medium (RPMI) supplemented with 10% FBS, 2 mM l-glutamine, penicillin (50 IU/mL), and streptomycin (50 mg/mL). Cells were maintained at 37 °C in a humidified atmosphere containing 5% CO_2_ and subcultured prior to confluence using trypsin/ethylenediaminetetraacetic acid (EDTA).

### Production of HFn in *Escherichia coli* and Purification

The production of HFn was carried out following a protocol set up in our lab.^32^ Briefly, a strain of *E. coli* BL21(DE3) was transformed using pET30b/HFn plasmid and grown at 37 °C in Luria Bertani Kanamycin (50 μg/mL) medium up to the point that OD_600nm_ = 0.6. Then, the cells were induced with 0.5 mM of isopropyl β-d-1-tiogalactopiranoside (IPTG) for 2 h and 30 min. After that, they were collected by centrifugation (4000*g*, 15 min), washed with phosphate-buffered saline (PBS), and resuspended in a lysis buffer with lysozyme and DNase I. After sonication, the crude extract was heated at 70 °C for 15 min and centrifuged. The supernatant was loaded onto DEAE Sepharose anion exchange resin, pre-equilibrated with a buffer containing 20 mM 2-(*N*-morpholino)ethanesulfonic acid potassium salt, pH 6.0. The elution of the purified protein was achieved by means of a stepwise NaCl gradient (from 70 to 420 mM). The fractions were analyzed by sodium dodecyl sulfate-polyacrylamide gel electrophoresis (SDS-PAGE) using 12% (v/v) polyacrylamide gels. Afterward, the purest fractions were collected together, and the buffer exchanged with PBS by means of several washings with Amicon centrifugal filters (100 kDa molecular weight cut-off (MWCO)). The concentration and any possible DNA contamination of the obtained solution were checked by measuring, respectively, the absorbance at 280 nm and the absorbance ratio 260/280, using a UV–vis spectrometer.

### Synthesis of Mn@HFn-RT and Mn@HFn-HT

HFn was washed with three volumes of *N*-(2-hydroxyethyl)piperazine-*N*′-ethanesulfonic acid (HEPES) 20 mM pH 7.5 using an Amicon filter (100 kDa MWCO) to exchange the protein buffer and concentrate the solution at 8–9 mg/mL. Then, a volume of a stock solution containing 2 mg of HFn was diluted to 1.44 mL with 1 M HEPES pH 8.25, and 60 μL of MnCl_2_ 450 mM were slowly added (6 μL every 10 min). During the latter step, the reaction temperature was set at 27 or 65 °C, generating NPs with different features, named, respectively, Mn@HFn-RT and Mn@HFn-HT. The solution was stirred at 80 rpm during the addition of MnCl_2_ and for the subsequent 2 h to promote ion internalization. Afterward, the obtained solution was centrifuged for 10 min at 3100*g* at 4 °C to remove any precipitate (denaturated HFn) from the sample. To eliminate the excess of Mn from the solution, the supernatant was cleaned up by means of centrifugal filtration (Amicon filter 100 kDa MWCO) and then passed through a Zeba Spin Desalting Column (7 kDa MWCO).

### Characterization of Mn@HFn-RT and Mn@HFn-HT

The protein was quantified by both measuring absorbance at 280 nm and using the Coomassie Plus Protein Assay Reagent (Thermo Fisher Scientific). The precise native structure was determined by native PAGE using 6% (v/v) polyacrylamide gels colored with Coomassie Blue staining. The hydrodynamic diameter was evaluated by dynamic light scattering (DLS) analysis using a Zetasizer Nano ZS ZEN3600 from Malvern Instruments Ltd. (Worcestershire, U.K.). The protein was diluted in PBS, 0.1 mg/mL. The measurement occurred at 25 °C by means of a HeNe 633 nm laser operating at 4 mW with a protein refractive index of 1.45 and using a scattering angle of 90°. A disposable cuvette with a 1 cm optical path length was used for the measurements. The results were expressed as a mean ± a standard deviation (SD) of three measurements. For transmission electron microscopy (TEM) analysis, an FEI 120 kV Tecnai G2 Spirit BioTWIN microscope with an accelerating voltage of 120 kV was used. Two microliters of the sample (0.5 mg/mL) were deposited on a Formvar-coated copper grid. After 5 min, excess solution on the grid was dried. The day after that, the sample was stained with uranyl acetate (1% in PBS, pH 7.5). The final dried sample was analyzed by TEM. The measurement of the inner and the outer diameter of the protein was obtained using ImageJ software by analyzing at least 150 particles of HFn from three different images. To quantify the concentration of Mn in the solutions, inductively coupled plasma optical emission spectroscopy (ICP-OES) Optima 7000 DV PerkinElmer was used: 100 μL of the sample (HFn = 0.1 mg/mL) was added to 0.5 mL of fresh aqua regia solution and left overnight. Thereafter, the solution was diluted with 2 mL of distilled water. Every sample was prepared and analyzed in triplicate. The results are expressed as a mean ± SD.

### Relaxivity Studies

The values of the longitudinal (*T*_1_) and transverse (*T*_2_) relaxation times of Mn@HFn-RT and Mn@HFn-HT were measured with a 0.47 T (20 MHz) time-domain NMR benchtop system (Bruker Minispec mq20), using, respectively, t1_sr_mb and t2_cp_mb sequences. The samples were analyzed using 200 μL of solution in 10 mm diameter NMR glass tubes and left in the relaxometer at 310 K. *R*_1_ and *R*_2_ relaxation rates (*R*) were calculated as 1000/*T*_1_ and 1000/*T*_2_, respectively, while *r*_1_ and *r*_2_ relaxivity (*r*) coincided with the angular coefficient of the calibration curve *R* against the Mn concentration. The *T*_1_-weighted phantom images of Mn@HFn were obtained using a 7 T MRI imaging system (PharmaScan, Bruker BioSpin, Billerica, MA). A multislice multiecho sequence with a repetition time of 0.4 s and an echo time of 10 ms was used. GdDTPA, a commercial contrast agent, was selected as a positive control. Solutions with the same concentration of Mn or with the same concentration of protein were compared, and a protein concentration equal to 0.1 mg/mL was chosen.

### Electron Spin Resonance (ESR) Characterization of Mn@HFn-RT

ESR studies on PBS solutions of Mn@HFn-RT and MnCl_2_ samples were carried out using a Bruker EMX spectrometer operating in X-Band, with a frequency modulation of 100 kHz, 10 mW of microwave power, magnetic field modulation of 5 Gauss, and equipped with an Oxford cryostat operating in a range of temperatures between 4 and 298 K. Spectra were acquired at 130 K.

### Kinetics of Mn Release In Vitro

This study was conducted introducing 1 mL of the sample (HEPES 20 mM pH 7.5 containing Mn@HFn-RT 0.75 mg/mL) in a dialysis bag (Float-A-Lyser, 100 kDa), which is then placed in a vessel containing 5 mL of the same buffer maintained under continuous stirring. At the desired time points (0 and 4, 18, 24, 48, and 72 h), 200 μL of dialysates were withdrawn and analyzed using the relaxometer Bruker Minispec mq20. The experiment was conducted in triplicate, and the results are expressed as a mean ± SD. The samples containing MnCl_2_ or MnCl_2_ + HFn were used as controls.

### Cellular Binding Assay by Flow Cytometry

Fluoresceine isothiocyanate (FITC)-Mn@HFn-RT was prepared by incubating 5 mg of Mn@HFn-RT (2 mg/mL) with 1 mg of fluoresceine isothiocyanate (FITC, Sigma) (2 mg/mL) in NaHCO_3_ 0.1 M for 2 h, subject to stirring. The solution was then passed through a Zeba Spin Desalting Column (7 kDa MWCO) to remove excess FITC. HCC1954 and NIH-3T3 cells (3 × 10^5^) were incubated for 45 min at 4 °C in flow cytometry tubes in the presence of 0.05 mg/mL FITC-Mn@HFn-RT by itself or with 1 mg/mL transferrin. After incubation, cells were washed three times with PBS. Labeled cells were resuspended with 0.3 mL of PBS-EDTA 2 mM and analyzed by CytoFLEX flow cytometry (Beckman Coulter). Typically, 1 × 10^4^ events were acquired for each analysis after gating on single cells, and a sample of untreated cells was used to set the appropriate gate on the region of positivity. The data reported the percentage of positive cells as a mean ± SD of three individual experiments. Untreated cells were used as a control.

### Cellular Uptake Assay by Flow Cytometry

HCC1954 and NIH-3T3 were cultured on 12-well plate up to the point of subconfluence and incubated with 0.1 mg/mL FITC-Mn@HFn-RT for different periods of time (1, 5, and 24 h). After two steps of washing with PBS, the cells were detached using trypsin/EDTA, washed three times by centrifugation with PBS, and finally resuspended with 0.3 mL of PBS-EDTA 2 mM. Samples were analyzed by CytoFLEX flow cytometry (Beckman Coulter). Typically, 1 × 10^4^ events were acquired for each analysis after gating on single cells, and a sample of untreated cells was used to set the appropriate gate on the region of positivity. The data reported the percentage of positive cells as a mean ± SD of three individual experiments. Untreated cells were used as a control.

### Cellular Uptake by ICP-OES

HCC1954 cells (1.5 × 10^6^) were plated and, on the day after plating, were incubated with 0.1 mg/mL of Mn@HFn-RT for 30 h. Then, cells were washed five times with PBS, detached using trypsin/EDTA, and counted. After a final step of washing with PBS, cells were collected, resuspended in 0.2 mL of PBS, and digested with 2 mL of aqua regia for 72 h. After the addition of a further 0.4 mL of distilled water, the samples were analyzed by ICP-OES Optima 7000 DV PerkinElmer. Each sample was measured three times, and the results are expressed as a mean ± SD.

### In Vivo MR Imaging

Six week old athymic nude-Foxn1nu mice (*n* = 6) were purchased by Envigo and maintained in a fully equipped facility, where their conditions were observed daily. All experiments were conducted under an approved protocol (authorization no. 994/2016-PR) and the animals handled according to the guidelines of the Italian Ministry of Health. HCC1954 cells were cultured as described above, and a 2:1 mixture of cell suspension (1 × 10^7^ cells) and Matrigel was implanted subcutaneously in each mouse. The animals were observed daily, their tumor size was measured with a caliper, and their weight was measured every 3 days. When the tumors reached a dimension of around 100–200 mm^3^, the mice were intravenously injected with a single dose of Mn@HFn-RT (50 mg/kg HFn corresponding to 1.2 mg/kg Mn), and MR images were acquired with a Bruker Pharmascan 7.0 T on animals anesthetized with isoflurane gas. The acquisitions were carried out before the injection (*n* = 6), and 1 h (*n* = 3), 5 h (*n* = 6), and 24 h (*n* = 3) after the injection. Then, images were analyzed using Paravision software, and the intensity values of a muscle region were used to normalize the intensity values of tumor regions. The final results are expressed as a mean ± standard error (SE).

### Statistical Analysis

Statistical analyses were conducted using the two-tailed Student’s *t*-test. The statistical significance is set as follows: *P < 0.05, **P < 0.01, ***P < 0.005, and ****P < 0.001.

## Results and Discussion

### Synthesis, Optimization, and Characterization of Mn-Loaded HFn

**Figure 1:**
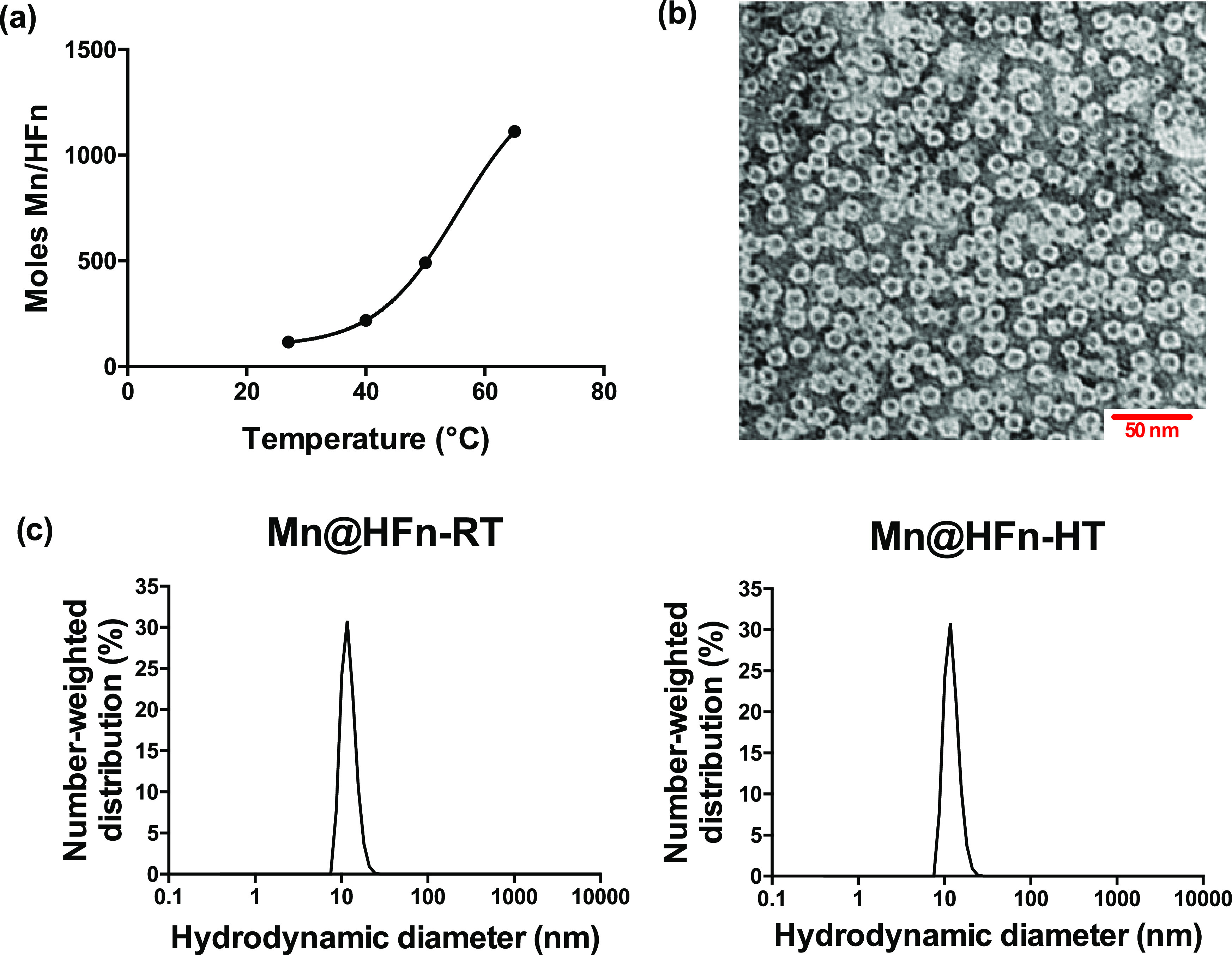
Characterization of Mn-loaded HFn: (a) correlation between Mn loading in HFn and reaction temperature, (b) TEM image of Mn@HFn-RT after negative staining, and (c) Mn@HFn-RT and Mn@HFn-HT size distribution detected by DLS analysis.

HFn was produced and purified, and the good quality of each batch was assessed according to a protocol established in our laboratory.^32^ The encapsulation of Mn ions was achieved by incubating MnCl_2_ with HFn under controlled conditions. Notably, the presence of a HEPES buffer prevented the precipitation of Mn and the pH level of 8.25 allowed Mn ions to maintain a state of low (II) oxidation. First, it was observed that the Mn loading, as evaluated by ICP-OES analysis, increased with the reaction temperature (Figure 1a). Thus, to optimize the features of the nanocomplex, we considered the effect of temperature on two different parameters: the Mn loading and the maintenance of spin active oxidation state. Although the enhancement of Mn encapsulation could be perceived as beneficial for improving the performance of the nanocomplex, elevated reaction temperatures may cause the metal to oxidize, thus decreasing its power as a CA.^33^ Differently from Gd, in which the active form is in its maximum oxidation state, the paramagnetic Mn(II) is in an intermediate state and inherently less stable. Two different preparations were generated, with the reaction temperature at 27 °C for Mn@HFn-RT and 65 °C for Mn@HFn-HT, and compared in terms of their physicochemical properties, MRI contrast efficiency, and effect on cell viability. These two batches were selected because, despite their different physical appearance (Figure S1a), the structural integrity of HFn was maintained in both, as shown by native gel electrophoresis (Figure S1b), TEM analysis after negative staining (Figure 1b), and DLS analysis (Figure 1c). This result is consistent with previous studies, in which HFn was found to maintain its structural integrity up to 80–90 °C.^34^

In this paragraph, the main results from the comparison between these two synthetic conditions are shown, while further details are reported in the Supporting Information. As shown in Table 1, the reaction yield (*n* = 5) was acceptable in both preparations, although a lower reproducibility was observed in the Mn@HFn-HT synthesis. Mn encapsulation was qualitatively confirmed by TEM analysis without negative staining (Figure S1c) and quantitatively assessed after protein disassembly by ICP-OES (Table 1). The *r*_1_ relaxivity (Table 1 and Figure S1d) was significantly higher in Mn@HFn-RT (28.3 ± 2.8 mM^–1^ s^–1^) as compared to Mn@HFn-HT (2.8 ± 0.5 mM^–1^ s^–1^), despite the fact that the Mn/HFn molar ratio was significantly increased in the Mn@HFn-HT batch. Surprisingly, Mn@HFn-RT proved to perform better than the approved Gd-based CAs analyzed under the same experimental conditions (e.g., Dotarem 3.4 mM^–1^ s^–1^; Primovist 5.3 mM^–1^ s^–1^).^35^ To investigate whether the discrepancy between Mn@HFn-HT and Mn@HFn-RT relaxivity was attributable to metal oxidation, a colorimetric assay^36^ was conducted to quantify the oxidized species of Mn. As shown in Figure S2a, a significant number of higher oxidation states, including Mn^3+^, Mn^4+^, and Mn^7+^, were detected in the Mn@HFn-HT sample (68 ± 14%). Moreover, additional experiments carried out with MnCl_2_ over time demonstrated that metal oxidation, as well as the lowering of relaxivity, was clearly correlated with an elevated reaction temperature (Figure S2b,c).

**Table 1:** Comparison between Mn@HFn-RT and Mn@HFn-HT in Terms of Reaction Recovery (%), Amount of Mn Encapsulated (as Evaluated by ICP-OES), and Relaxivity Properties (*r*_1_ and *r*_2_/*r*_1_)^a^

	Mn@HFn-RT	Mn@HFn-HT
HFn recovery rate (%)	70 ± 4	56 ± 12
Mn/HFn molar ratio	218 ± 33	1087 ± 173
*r*_1_ (mM^–1^ s^–1^)	28.3 ± 2.8	2.8 ± 0.5
*r*_2_/*r*_1_	2.3 ± 0.3	2.9 ± 0.4

a Data are expressed as the mean of five replicates ± SD.

**Figure 2:**
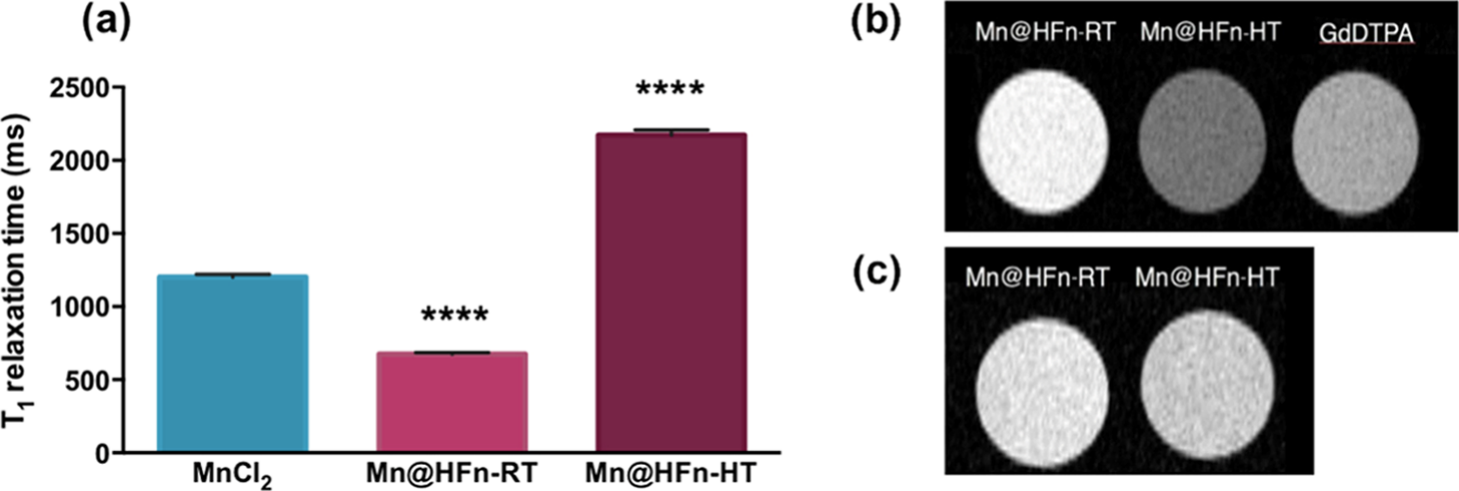
(a) *T*_1_ relaxation time of three different solutions containing the same amount of Mn ions as measured with a 0.47 T NMR relaxometer. Reported values are a mean of three replicates ± SD. ****P < 0.001 (Student’s *t*-test) vs MnCl_2_, (b) *T*_1_-weighted images of Mn@HFn-RT, Mn@HFn-HT, and GdDTPA containing the same concentration of metal (0.1 mM), and (c) *T*_1_-weighted images of Mn@HFn-RT and Mn@HFn-HT containing the same concentration of HFn (0.1 mg/mL).

Afterward, the protein loading contribution to the relaxation was investigated by comparing the behavior of encapsulated (either Mn@HFn-RT or Mn@HFn-HT) vs free Mn ions (MnCl_2_) in solutions containing the same amount of metal. As shown in Figure 2a, a variation in the relaxation time was detected in both HFn samples. Notably, Mn oxidation negatively affected the relaxivity of Mn@HFn-HT, whereas the Mn entrapment in Mn@HFn-RT induced a substantial decrease in the *T*_1_ value. The improved performance of the latter sample can be explained assuming that the metal complexation induced a different interaction between Mn^2+^ and water; indeed, it has been reported that CA dynamics significantly affect their relaxivity.^37^ For this reason, protein binding to paramagnetic metal ions was exploited to improve CA efficiency by slowing down molecular tumbling and promoting a fast rate of exchange of coordinated water molecules.^38^

Accordingly, the *T*_1_-weighted images of Mn@HFn-RT acquired with a 7 T MRI imaging system displayed the brightest signal as compared to both Mn@HFn-HT and the clinically approved GdDTPA at the same concentration of metal (0.1 mM, Figure 2b). However, this experiment and the above-collected data were not sufficient to establish the superiority of Mn@HFn-RT over Mn@HFn-HT in terms of CA power. Since the injectable protein amount is the limiting factor for in vivo applications, it was also useful to compare these CAs in terms of HFn concentration rather than Mn ion dosage. Thus, the latter experiment was repeated by normalizing the sample with respect to HFn concentration. As reported in Figure 2c, under this condition, Mn@HFn-RT and Mn@HFn-HT were almost equivalent in brightness. This probably occurred because the lower relaxivity power of Mn in Mn@HFn-HT was balanced out by the higher encapsulation efficiency of this formulation. Indeed, despite the Mn oxidation, the absolute amount of Mn(II) in Mn@HFn-RT and Mn@HFn-HT was comparable, and hence, their contrast ability was reasonably similar.

Finally, the effect of HFn-based CAs on cellular viability was assessed in two TfR1-expressing cells (HeLa and HCC1954, Figure S3). As summarized in Figure S4a,b, Mn@HFn-RT affected cellular metabolic activity only slightly, while Mn@HFn-HT displayed significant toxicity at all time points tested. These experiments also suggested that the elevated metal concentration could be the cause of the alteration observed in the high-temperature preparation.

Altogether, these results highlighted that, despite the fact that the encapsulation reaction conducted at a higher temperature increased the Mn ion loading efficacy significantly, Mn@HFn-HT did not improve the contrast power as compared to Mn@HFn-RT while exhibiting more pronounced negative implications on cell viability. Therefore, Mn@HFn-RT was selected as the most promising CA and the subsequent experiments were thus conducted with this nanocomplex.

### Assessment of Mn Complexation in Mn@HFn-RT

**Figure 3:**
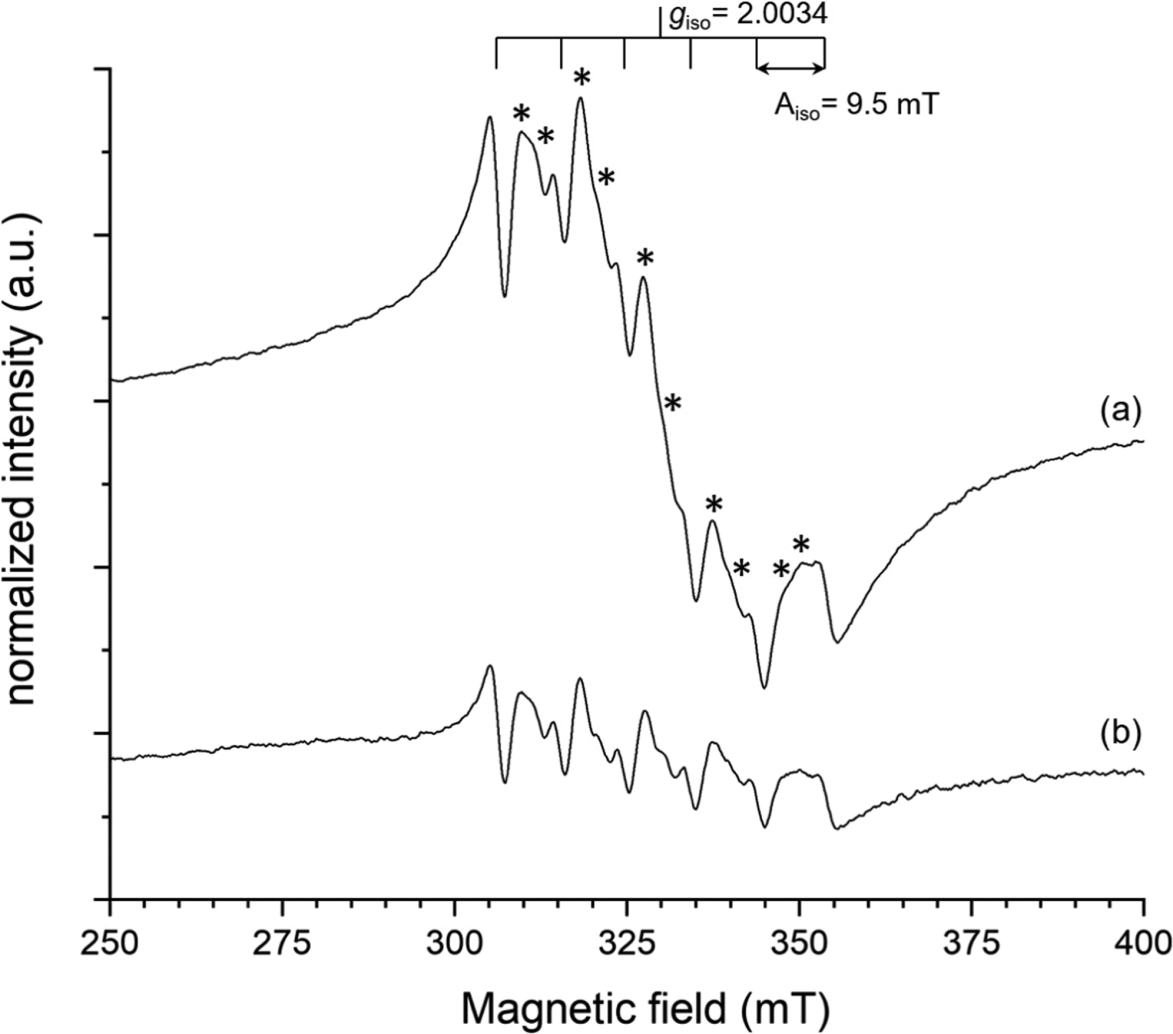
ESR spectra at 130 K of (a) Mn@HFn-RT and (b) MnCl_2_ dispersed in PBS. Forbidden transitions are indicated with *.

The Mn(II) binding to HFn was confirmed by ESR. Figure 3 shows the spectra obtained at 130 K for PBS solutions of Mn@HFn-RT (spectrum a) and MnCl_2_ (spectrum b) enclosing comparable concentrations of Mn(II) ions.

Both the samples exhibit a relatively sharp centered sextet attributed to the hyperfine-split central transition (|+1/2⟩ ↔ |−1/2⟩) of high-spin (*S* = 5/2) ^55^Mn(II) complexes.^39^ No signals from oxidized Mn species were detected. The *g*_iso_ value was measured as 2.0034, which is very close to that for free electrons, suggesting the absence of spin–orbit coupling in the ground state. The measured values of main line splitting from the low to high field (8.9, 9.3, 9.5, 9.8, 10.1 mT) are in good agreement with those reported in the literature for d^5^ high-spin Mn(II) complexes.^40^

Between each two main hyperfine lines, a pair of low-intensity resonances with an average spacing of 2.3 mT corresponding to formally forbidden transitions (Δ*m*_I_ = ±1) is detectable for both Mn@HFn-RT and MnCl_2_ (starred resonances in Figure 3). These lines are related to the further intermix of nuclear hyperfine levels operated by the zero-field splitting (ZFS) parameters of the Hamiltonian.^39^ In the present case, ZFS can be referred to as lowering from cubic (or higher) symmetry of the Mn(II) ion in the complex, due to tetragonal distortions or changes in the bonding strength as an effect of the modification of the crystal-field.

Besides these similarities, a closer inspection reveals significant differences in the breadth and intensity of several spectral features. In particular, signals in Mn@HFn-RT appear more intense than in MnCl_2_, mainly due to the presence of a broader component superimposing to the sextet lines in the spectrum. This may be attributed to an enhanced spin–lattice relaxation due to dipolar interaction and random orientation of Mn(II) species when encapsulated in the protein.^41^

Interestingly, the intensity of the forbidden transitions (*I*_forb_) compared to the main hyperfine transitions (*I*_allowed_) is higher in the spectrum of Mn@HFn-RT than in MnCl_2_ (Figure 3). As already introduced, the appearance of these prohibited transitions is relatable to the magnitude of ZFS (in particular to the factor *D*) and to the electron Zeeman splitting. In detail, the following relation can be invoked^39^
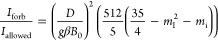


Thus, it can be qualitatively inferred that the increased *I*_forb_/*I*_allowed_ ratio observed for Mn@HFn-RT entails a largest axial ZFS parameter, supporting a change in the symmetry of Mn(II) ions as a result of their effective complexation by HFn.

Altogether, ESR data confirmed that Mn(II) ions incorporated in HFn are actually complexed by chelating residues of the protein rather than merely entrapped in the inner cavity. To confirm that the amount of the aspecifically and weakly bound Mn is low, Mn@HFn-RT was incubated with EDTA (1:1 molar ratio with Mn encapsulated) and *T*_1_ value of this solution compared with several controls (Figure S5). Although a decrease in HFn@Mn-RT relaxivity was observed after EDTA addition, the recorded *T*_1_ value is not as high as for the samples co-incubating free Mn + EDTA ± HFn. This further demonstrates that most of the Mn is stably complexed in the inner core of HFn; otherwise, the metal should be chelated by EDTA strongly decreasing its contrast power.

### Study of the Stability of the Contrast Power of Mn@HFn-RT

**Figure 4:**
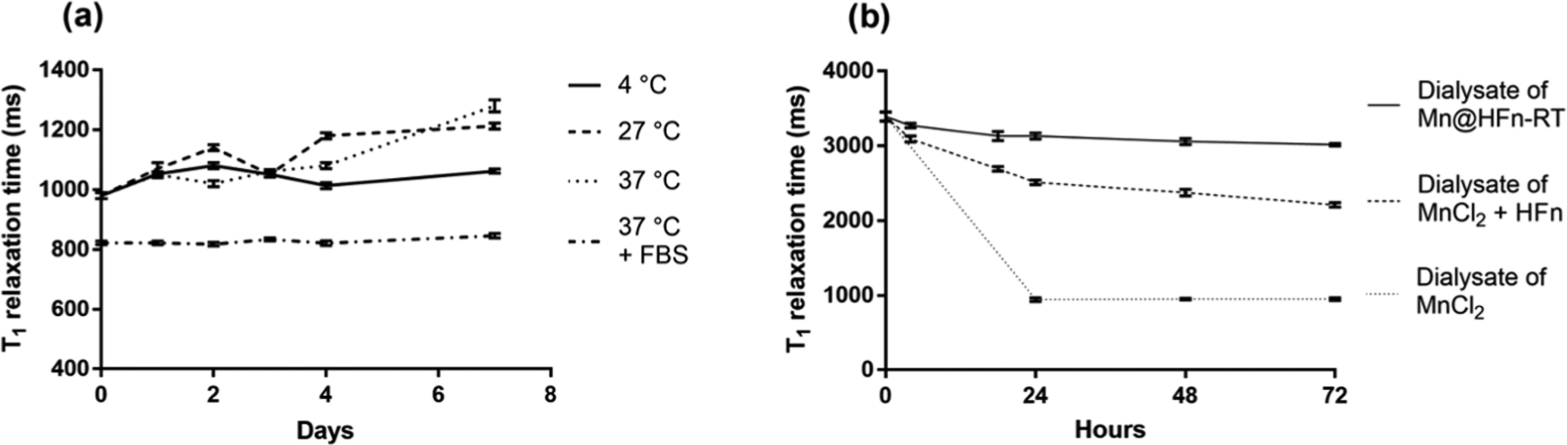
(a) *T*_1_ relaxation time measured with a 0.47 T NMR relaxometer on Mn@HFn-RT samples left for 1 week at 4, 27, 37, and 37 °C in the presence of FBS 10%. Each point is the average of two samples measured twice and (b) the results of a Mn release test in HEPES buffer 20 mM performed with a 0.47 T NMR relaxometer. Dialysates of MnCl_2_, Mn@HFn-RT, and MnCl_2_ + HFn were checked, and the *T*_1_ relaxation time was expressed as a function of time. Each point is the average of four measurements.

Mn@HFn-RT stability in an aqueous solution was checked under different incubation conditions. Aliquots were kept at 4, 27, 37, and 37 °C with the addition of 10% FBS, and the relaxation time was monitored every day for 1 week (Figure 4a). Incubation at 4 °C proved to be the best option for maintaining the Mn@HFn-RT relaxivity unaltered. Increased temperatures contributed to slightly enhancing the relaxation time, thus decreasing the nanocomplex relaxivity. Interestingly, when kept at 37 °C in the presence of FBS, the magnetic properties of the nanocomplex were more robust and the relaxation power was stably maintained for a week. It should be mentioned that the lower value observed at the first point in time of samples incubating in the presence of FBS was due to the different medium rather than to a change in the nanocomplex relaxivity; indeed, the *T*_1_ relaxation time of the HEPES buffer with and without FBS was 3370 ± 70 and 3730 ± 80 ms, respectively. Overall, these data suggest that the Mn nanoformulation was moderately stable, a conclusion that was further confirmed when the storage conditions were investigated. Indeed, after 3 weeks at 4 or −20 °C, the relaxation time of the encapsulated Mn was not significantly affected (Figure S6). Finally, a study was conducted to assess the possible release of Mn from Mn@HFn-RT. Considering the issues experienced in separating free from encapsulated Mn, the experiment was performed by exploiting the dialysis principle. At predetermined time points, aliquots of the dialysate (the buffer outside the dialysis membrane) were withdrawn and the *T*_1_ relaxation time was measured. The decrease of the latter value is an indirect indication that Mn passed through the dialysis membrane after being released by HFn. To rule out any interference caused by the interaction between the protein and the free metal ions, a solution containing the same number of moles of HFn and Mn^2+^ was used as a control (MnCl_2_ + HFn).

As reported in Figure 4b, the two dialysates exhibited a significant difference within a few hours, and the gap was even more evident after 24 h, at which point the relaxation time of the Mn@HFn-RT dialysate was 3130 ± 40 ms, while in the MnCl_2_ + HFn dialysate, it decreased to 2510 ± 30 ms. Although we observed an initial slight decrease, the *T*_1_ relaxation time of the Mn@HFn-RT dialysate was consistent over time, whereas in the MnCl_2_ + HFn sample, the downward trend was more pronounced and continuous. Taken together, the *T*_1_ relaxation time of Mn@HFn-RT did not change substantially because the metal was unable to move freely, suggesting an effective and stable entrapment inside the core of the protein. However, it should be noted that the decrease in the *T*_1_ relaxation time of MnCl_2_ + HFn dialysate was not as high as observed when MnCl_2_ was tested (for about ∼950 ms), indicating that the protein was able to interact with the metal ions to some extent.

### In Vitro Cellular Binding and Uptake of Mn@HFn-RT

**Figure 5:**
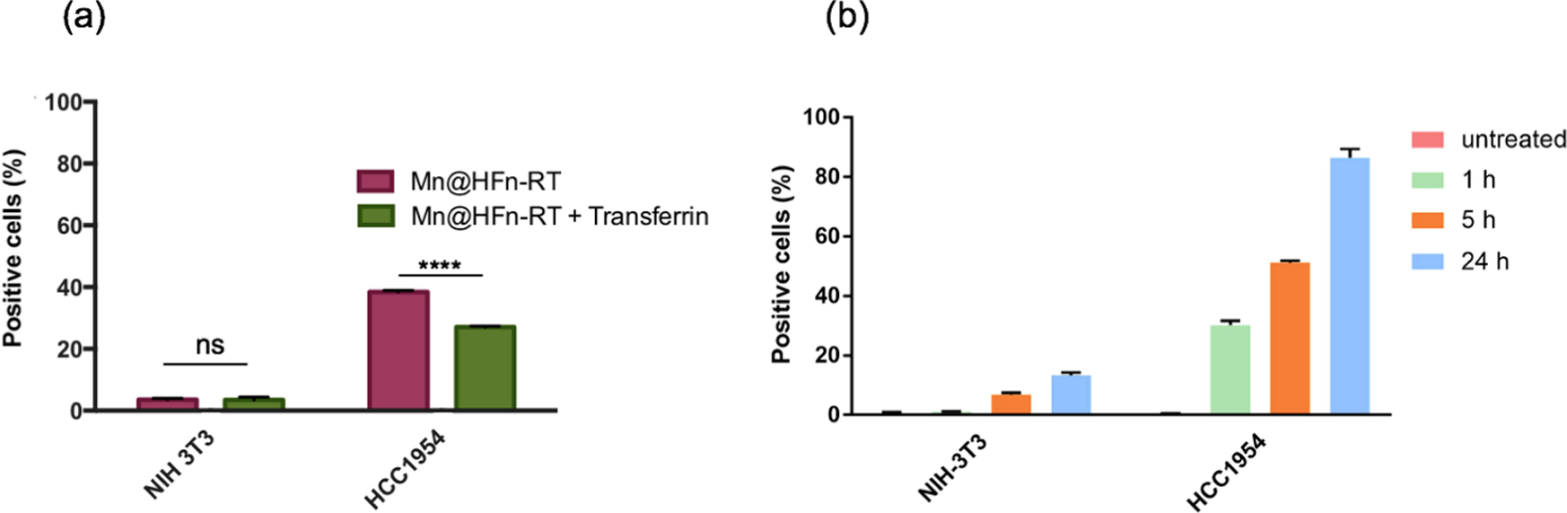
(a) NIH-3T3 and HCC1954 cells were incubated with FITC-Mn@HFn-RT (0.05 mg/mL) with and without transferrin (1 mg/mL) for 45 min at 4 °C and then processed with flow cytometry (*n* = 3). The data represent the percentages of cells in the positive region; the values are the mean ± SD (*n* = 3). ****P < 0.001 (Student’s *t*-test) and (b) NIH-3T3 and HCC1954 cells were incubated with FITC-Mn@HFn-RT (0.1 mg/mL) at different time points at 37 °C and then processed with flow cytometry (*n* = 3). The data represent the percentages of cells in the positive region; the values are the mean ± SD (*n* = 3).

The data presented above demonstrated that our Mn-based contrast enhancer showed superior contrast ability compared to already-approved products and long-term stability in physiological solutions. The next objective was to establish the capability of Mn@HFn-RT to be internalized selectively by TfR1-positive cells. After demonstrating that all of the concentrations next tested (below 0.1 mg/mL) do not affect the cell viability (Figure S7), we performed a flow cytometry analysis whose primary purpose was to investigate its binding efficiency (Figure 5a). Mn@HFn-RT labeled with FITC (FITC-Mn@HFn-RT) was incubated at 4 °C for 45 min with either HCC1954 cells or the TfR1_low_ cell line (NIH-3T3) used as a negative control.^42^ The experiment was conducted with FITC-Mn@HFn-RT alone or in the presence of the competitor transferrin (Tf). The nanocomplex concentration was adjusted to avoid reaching 100% positive cells in HCC1954 samples so that we could better appreciate the difference between the cell lines. The results revealed that the nanocomplex bound to the cell membrane was significantly higher in HCC1954 cells (38.4%) as compared to NIH-3T3 cells (3.5%) and that in HCC1954 samples the binding capability significantly decreased when the cells were coincubated with Tf (27%), indicating that TfR1 played a fundamental role in the process. However, an incomplete inhibition was observed with Tf, likely attributable to the fast recycling of TfR1.^43^ In addition, we speculate that multivalent HFn had a greater chance of binding to TfR1 as compared to the monovalent Tf.^44^

Next, another experiment was performed to measure the uptake of FITC-Mn@HFn-RT; this experiment involved incubating the nanocomplex at 37 °C with seeded cells and analyzing them at different time points (1, 5, and 24 h) by flow cytometry. The results shown in Figure 5b confirmed the selective uptake of Mn@HFn-RT by cells overexpressing the TfR1 receptor (i.e., HCC1954 cells), as well as the time dependence of the process. Since the flow cytometry signal may indicate both cellular binding and internalization, the same experiment was conducted with confocal microscopy, and the uptake of FITC-Mn@HFn-RT was confirmed (Figure S8). To prove that the protein was also able to bring the metal content inside the cells, an ICP-OES experiment measured the amount of Mn in HCC1954 cells incubated with Mn@HFn-RT for 30 h. Despite the intracellular level of Mn being carefully controlled and the possibility that it does not precisely reflect the actual amount of delivered metal, the Mn content found in the treated sample (11.6 ng of Mn/million cells ± 0.72) was 7.3 times higher than the value recorded for untreated cells (1.58 ng of Mn/million cells ± 0.19), suggesting a substantial intake of Mn ions upon cell exposure to Mn@HFn-RT.

### In Vivo Imaging of Cancer Lesions with Mn@HFn-RT

**Figure 6:**
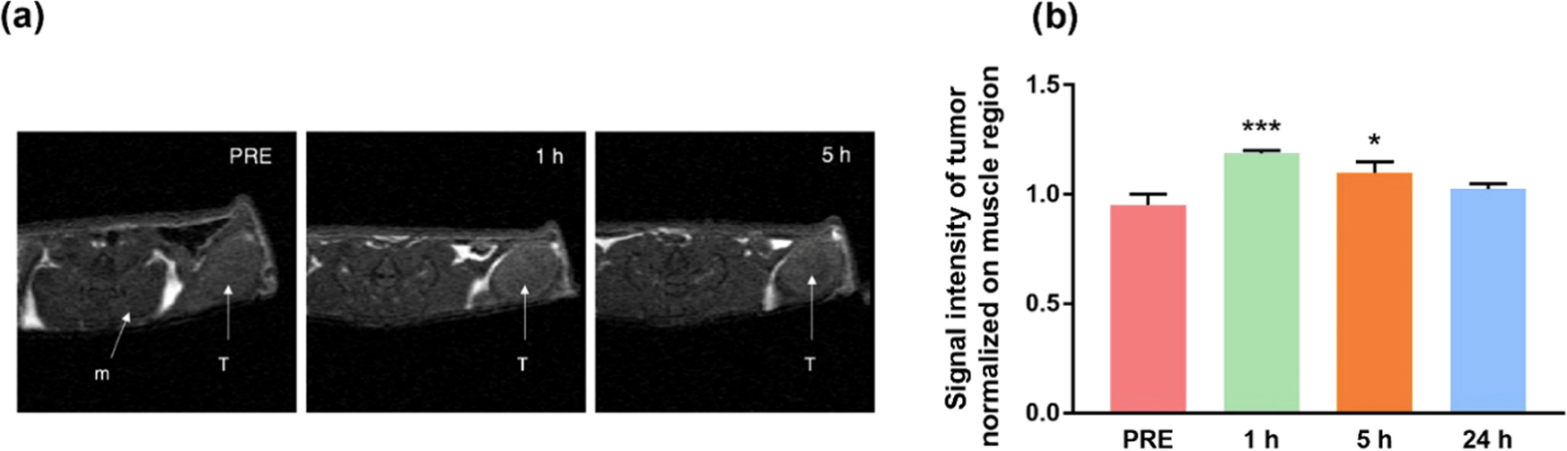
(a) Representative *T*_1_-weighted MRI images of a mouse obtained before (PRE) and after (1 and 5 h) the injection of Mn@HFn-RT (50 mg/kg HFn). The arrows indicate the muscle (m) and tumor (T) regions and (b) the intensity of the bright signal quantified in the tumor region normalized against the brightness of the muscle. The values are the mean of at least three mice ± SE. *P < 0.05; ***P < 0.005 (Student’s *t*-test).

The encouraging results obtained in vitro have enabled to test the Mn@HFn-RT CA efficacy in vivo. To this aim, HCC1954 breast cancer cells were subcutaneously inoculated into six nude mice. When the tumors reached a size in the range 100–200 mm^3^, the mice were intravenously injected with Mn@HFn-RT (protein dosage of 50 mg/kg of HFn; 1.2 mg/kg of Mn) and *T*_1_-weighted images were obtained at predetermined time points (preinjection and 1, 5, and 24 h postinjection). The signal intensity of the tumor region was measured, normalized against the signal intensity detected in a proximal muscle, and the brightness variation was evaluated vs the images captured before the treatment (PRE). As highlighted by the representative images in Figure 6a, tumor masses and their borders were well-defined and recognizable, especially 1 h after the administration of Mn@HFn-RT. The visual evidence was further confirmed by the quantification of signals (Figure 6b) with a significant enhancement (+25% vs the preinjection value) being observed at the first point in time and the tumor brightness progressively decreasing with time, becoming negligible at 24 h. These results suggest that HFn was efficiently accumulated in the tumor region and that Mn was able to produce a hyperintense signal, facilitating the identification of cancerous lesions. The effectiveness of Mn@HFn-RT CA was even more surprising considering the modest amount of Mn injected (1.2 mg/kg) and the fact that the enhancement related to a tumoral tissue. Indeed, as reported in literature for Mn-based nano-CAs, dosages comparable to Mn@HFn-RT (0.5–3 mg/kg Mn) were used to monitor the efficiency of CAs in clearance organs (liver, kidney, and bladder).^45−47^ When the contrast enhancement was checked in tumor regions, the administered doses required to achieve a clearly detectable signal were usually about 5–7 times higher.^48^ For example, Chen et al. reported a normalized signal intensity in glioma-bearing mice equal to ∼70% after the injection of MnO-TETT-FA NPs (8 mg/kg Mn).^49^ Also, Mi et al. designed PEGMnCaP NPs able to produce an efficient signal intensity (+60%) using a Mn administration 10 times superior to our experiment.^50^ Two MRI-in vivo studies in which the dosages reached 25 mg/kg Mn were also conducted to achieve a tumor signal intensity of up to 30% in the first^51^ and 40% in the second.^52^ Our enhancement was around 25% but was correlated with a metal administration that was 20 times lower and came with predictably much higher CA safety. Indeed, although clinical studies have not shown the toxic effects of exposure to Mn CAs, high concentrations of Mn are expected to cause side effects.^53^ The unusually high efficiency of Mn@HFn-RT in enhancing tumor contrast compared to conventional CAs could be attributable to (a) the particular structural features of our nanocomplex, including the elevated intrinsic relaxivity (28.3 mM^–1^ s^–1^), which allows relatively few Mn atoms to produce a strong relaxation process; (b) the dimensions of the nanocomposite (∼12 nm), which are favorable for promoting enhanced permeability and retention (EPR) in the process of passive accumulation in the tumor region;^54^ and (c) the high affinity of HFn for the TfR1 receptor, which fosters active targeting of cancer cells.^31^

**Figure 7:**
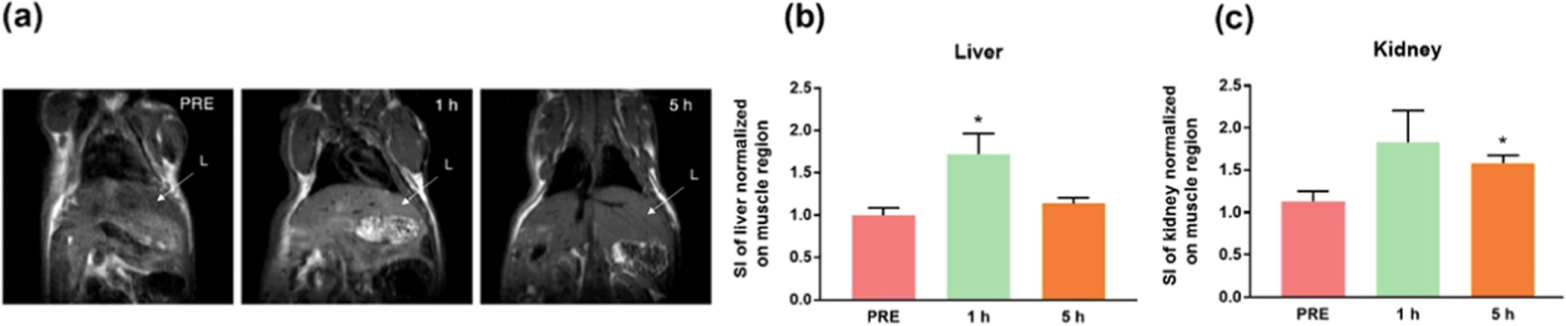
(a) Representative *T*_1_-weighted MRI images of a mouse obtained before (PRE) and after (1 and 5 h), the injection of Mn@HFn-RT. The arrows indicate the liver (L), (b) intensity of the bright signal quantified in the liver normalized against the brightness of the muscle ± SE. *P < 0.05 (Student’s *t*-test). (c) intensity of the bright signal quantified in the kidney normalized against the brightness of the muscle ± SE. *P < 0.05 (Student’s *t*-test).

In a previous work,^55^ biodistribution studies conducted using a different tumor model revealed that HFn was mainly cleared by the liver and kidneys, and for this reason, Mn@HFn-RT contrast was also monitored in these organs. Notably, in the liver (Figure 7a,b), after an initial strong contrast enhancement (ca. +60% vs PRE) at 1 h postinjection, the signal intensity drastically decreased at 5 h (+20% vs PRE), while in the kidneys (Figure 7c), the enhancement was maintained at the same levels over time (around +40% at 5 h vs PRE). Both results suggest that Mn@HFn-RT was progressively eliminated, and it is likely that immediately after its administration, the nanocarrier was mainly captured by the mononuclear phagocyte system, while at longer circulation times, it was primarily excreted in urine. These data were confirmed by a biodistribution experiment performed in an HCC1954 tumor model exposed to Alexa660-labeled HFn (AF660-HFn): in vivo epifluorescence analysis of the bladder monitored at predetermined time points after an AF660-HFn i.v. injection showed a strong fluorescence at 1 h, which decreased and became negligible after 24 h (Figure S9). The timing supported the investigation being conducted at an early point in time in the MRI experiment, in which the peak of intensity was observed after 1 h. Moreover, organs dissected from mice were analyzed ex vivo and quantified by measuring their epifluorescence intensity (Figure S10); the major accumulation was observed in the liver, with a discrete signal found also in the spleen and kidneys.

We emphasize that further experiments should be conducted to definitively rule out the possible toxicity of Mn@HFn-RT in vivo; nevertheless, there is evidence to support our hypothesis that this nanocomplex avoids important safety concerns. First, the nanocarrier itself consists of a self-assembly of multiple subunits derived from a protein already present in the human body and thus is expected to bypass recognition by the immune system and not trigger an inflammatory response. Second, Mn, a natural cellular constituent, is administered at very low dosages (around 1 order below the common dosages utilized for in vivo imaging of solid tumors). Finally, the rapid clearance of Mn@HFn-RT ensures an efficient excretion, also limiting possible time-dependent Mn release from the carrier, which is thought to be the main cause of side effects in conventional gadolinium-based CAs.

## Conclusions

In summary, we have investigated possible routes for obtaining a powerful and nontoxic Mn-based CA for MRI that is specifically designed to detect malignant lesions with strong selectivity and higher sensitivity compared to most Mn- and Gd-based CAs reported to date. A straightforward and facile protocol of the biomineralization of Mn ions inside the core of an HFn protein was set up at two different incubation temperatures (room temperature and 65 °C), and the products were characterized in terms of loading, undesired release, relaxivity, colloidal and magnetic stability, and their ability to affect human cell viability. The use of HFn as an excellent biocomplexation template was justified by (1) the natural tendency of apoferritin to promote metal(II) biomineralization and (2) the spontaneous tumor tropism of HFn. Despite its lower encapsulation efficiency, Mn@HFn-RT was selected for further investigations because, when tested at equal HFn concentrations, it proved to be able to enhance the signal brightness at the same level of the nanocomplex generated at 65 °C (Mn@HFn-HT) without substantially affecting cellular metabolic activity. Mn@HFn-RT demonstrated to stably complex Mn(II) ions in the inner cavity of the protein and showed a superior contrast capability as compared to free ions, associated with a faster relaxation of water molecules. In vitro experiments confirmed its ability to be bound and internalized by TfR1-positive cells. The strong contrast enhancement of Mn@HFn-RT, together with an expected improvement in tumor targeting due to HFn, suggests that this Mn nanocomplex could be developed for the *T*_1_-weighted MRI imaging of solid tumors. The abovementioned results along with the higher relaxivity displayed by our nanoformulation as compared to the conventional Gd-based CAs encouraged us to explore the potential of Mn@HFn-RT for in vivo experiments. It is noteworthy that the images obtained after injection of Mn@HFn-RT highlighted a well-defined region corresponding to the tumor mass. Altogether, the results of this study offer a new insight into the potential of Mn@HFn CAs. Specific advantages of our Mn@HFn-RT include (1) the use of Mn(II) ions stably complexed with HFn rather than in the form of MnO crystals; (2) the accumulation of several Mn(II) ions in a confined small area allows enhanced contrast from adjacent water molecules; (3) the observed contrast power was equal or higher than Gd enhancers; (4) this study represents the first example of Mn@HFn promoted targeted contrast enhancement at low metal doses in a nonclearance organ tumor mode. Indeed, the results indicated that a good contrast was observed with small doses of Mn, presumably minimizing possible clinical side effects. MRI experiments and biodistribution studies showed a very rapid clearance of HFn, suggesting that the best output could be achieved with an early acquisition of images. This rapid contrast effect could help both patients and physicians avoid long waiting times, making Mn@HFn-RT a promising nano-CA for application in clinical practice.

## Supporting Information Available

Experimental details, confocal and epifluorescence images, native polyacrylamide gel, relaxometric analyses, TfR1 cellular expression, and viability assays (PDF)
