# Supplementary material for: Development of an Effective Tumor-Targeted Contrast Agent for Magnetic Resonance Imaging Based on Mn/H-Ferritin Nanocomplexes
Source: ACS Appl Bio Mater. 2021 Oct 19;4(11):7800–10. doi: 10.1021/acsabm.1c00724 (PMC8596607; doi:10.1021/acsabm.1c00724)
Supplement: Supplementary file 1 — mt1c00724_si_001.pdf [file mt1c00724_si_001.pdf]

SUPPORTING INFORMATIONS

Development of an effective tumor-targeted contrast agent for magnetic resonance imaging based on Mn/H-ferritin nanocomplexes

Chiara Tullio[§,1] *Lucia Salvioni*[§,1] *Michela Bellini,*[1] *Anna Degrassi,*[2] *Luisa Fiandra,*[1] *Massimiliano D'Arienzo,*[3] *Stefania Garbujo,*[1] *Rany Rotem,*[1] *Filippo Testa,*[1] *Davide Prosperi,*[1,]* *Miriam Colombo*[1,]*

[1]NanoBioLab, Department of Biotechnology and Bioscience, University of Milano-Bicocca, Piazza della Scienza 2, 20126 Milano, Italy. [2]Preclinical Development, Efficacy and Safety, Accelera S.R.L. – NMS Group S.p.A., viale Pasteur 10, 20014 Nerviano (MI), Italy. [3]Department of Materials Science, University of Milano-Bicocca, Via Roberto Cozzi 55, 20125 Milano, Italy.

[§]equally contributed,

* corresponding authors: miriam.colombo@unimib.it; davide.prosperi@unimib.it.

Methods

Mn-oxidizing (leucoberbeline) assay. A solution of 0.04% leucoberbeline blue dye (Sigma Aldrich) in 45 mM acetic acid (Sigma) was used to quantify oxidized Mn (Mn(III), (IV) and (VII)), as reported in literature.[1] 500 µL of the mixture were added to 100 µL of Mn@HFn-RT and Mn@HFn-HT solutions, and after 30 min the absorbance was detected with a UV-vis spectrometer at 620 nm. Standard curves were obtained from $KMnO_4$ samples (Merk).

Cell viability assay. HCC1954 cells were seeded on a 96-multiwell dish with a density of 1 × 10^4 cells/well one day before the experiment. Then, cells were incubated with Mn@HFn-RT or Mn@HFn-HT, using solutions of $MnCl_2$ and HFn as controls. At the experimental timepoints, the medium was changed, and the cells incubated for 4 h at 37 °C with 3-(4,5-dimethyl-2-thiazolyl)-2,5-

diphenyl-2H-tetrazolium bromide (MTT) stock solution previously diluted in medium without phenol red following the manufacturer's instructions (Promega). After incubation, 0.1 mL of MTT solubilizing solution was added to each well to solubilize the MTT formazan crystals. Absorbance was read using a testing wavelength of 570 nm and a reference wavelength of 630 nm. The results are expressed as mean ± standard deviation (SD) of viability percentage calculated versus the untreated sample (n = 4-6).

TfR1 expression. 3 × 10^5 cells were immunodecorated in flow cytometry tubes with anti-TfR1 antibody (3 µg/tube; ADG3937, Sekisui, MA, USA) in phosphate buffered saline (PBS), 1% Bovine Serum Albumin (BSA; Sigma) for 30 min at RT. Then, cells were washed thrice with PBS and immunodecorated with Alexa Fluor 647 goat anti-mouse secondary antibody (3 µg/tube; A-21235 Thermo Fisher Scientific, MA, USA) in PBS, 2% BSA and 2% goat serum for 30 min at RT. After three washes with PBS, cells were analyzed by flow cytometry. 10^4 events were acquired for each analysis, after gating on viable cells and on singlets. The data (median fluorescence intensity) were normalized against the untreated sample and expressed as mean ± SD of 3 independent replicates. Cells immunodecorated with the secondary antibody only were used as control.

Cellular uptake by immunodecoration and confocal detection. 3 × 10^5 HCC1954 cells were cultured on cover glass slips precoated with polylysine and, after 24 h, incubated with 0.1 mg/mL of FITC-Mn@HFn-RT suspended in complete medium. Then, cells were washed PBS, fixed with 4% paraformaldehyde (37 °C, 20 min) and then subjected to membrane and nucleus staining by incubation with Wheat Germ Agglutinin (WGA)-Alexa Fluor 555 conjugate (1 µg/mL) and 4',6-diamidin-2-phenylindole (DAPI) (1 µg/mL), respectively. After washing with PBS, cover slips were mounted with ProLong Antifade reagent (Thermo Fisher Scientific, MA, USA) and examined by Nikon A1 Confocal Microscope (Nikon Instruments) equipped with laser excitation lines 405, 488 and 555 nm. Images were acquired at 1024 × 1024 pixel resolution and with a 63 × magnification oil-immersion lens. In order to confirm the correct setup of FITC channel, an untreated sample was as well analyzed, and no signal was found.

Epifluorescence analysis of AF660-HFn performed in vivo and ex vivo. HCC1954 cells were cultured and implanted subcutaneously in four nude mice in a mixture of 1×10^7 cells suspended in growth media and Matrigel. Animals were monitored and treated as described in Materials and Method section, until the tumor reached a dimension around 100-200 mm^3. To observe the biodistribution of HFn in mice body, the protein was labeled with Alexa Fluor™ 660 NHS Ester (AF660-HFn) and injected in the tail vein of mice (5 mg/kg HFn). PBS was used as control. Fluorescent images were obtained by placing the animals previously anesthetized with isoflurane gas in an IVIS Lumina II imaging system (Perkin Elmer) at 37 °C. The in vivo images were acquired after the nanoparticle injection (1, 4 and 24 hours) with a 720 nm emission filter, and excitation was scanned from 570 to 640 nm. Mice autofluorescence was removed by spectral unmixing. At 4 hours, mice were sacrificed, and organs were dissected and analyzed in the IVIS system. All the epifluorescence (Epf) intensity values were normalized after subtracting Epf values obtained by mice injected with PBS.

Discussion

Extensive physico-chemical characterization of Mn@HFn-RT and Mn@HFn-HT. Mn-loaded HFn were prepared following the protocols reported in the main text either at 27 °C (Mn@HFn-RT) and 65 °C (Mn@HFn-HT). After the purification, Mn@HFn-RT appeared as a transparent solution, while Mn@HFn-HT revealed a light brown color (Figure S1a). The protein recovery efficiency was 70% (± 4%) for Mn@HFn-RT, while a poor reproducibility along with a lower efficiency (56% (± 12%)) was observed when the reaction temperature was increased to 65 °C (Table 1).

Native gel electrophoresis showed that the protein maintained the original structure both in Mn@HFn-RT and Mn@HFn-HT samples (Figure S1b), while the hydrodynamic diameter assessed by DLS analysis (12.2 ± 0.5 nm and 13.5 ± 1.1 nm (Figure 1c), respectively) was consistent with the size reported for native ferritin (~12 nm).[2] TEM analysis confirmed that the protein preserved its distinctive structure after the reaction: indeed, core-shell architecture was clearly detectable with an

inner and outer effective diameter of 7.4 ± 2.2 nm and 12.1 ± 1.4 nm (Figure 1b). These results suggest that, in both samples, the maintained structural integrity of HFn should be able to promote the effective recognition of TfR1 receptor and the consequent cellular uptake.

A first evidence of Mn encapsulation in HFn was given by TEM analysis conducted in absence of the negative staining (Figure S1c): thanks to the metal complexation, it was possible to focus the protein cages in Mn@HFn-RT and Mn@HFn-HT samples. After protein disassembly in acidic environment, Mn was quantified by ICP-OES analysis and the Mn/HFn molar ratio was estimated as 218 (± 33) for Mn@HFn-RT and 1087 (± 173) for Mn@HFn-HT (Table 1).

Then, the relaxivity (r) of Mn@HFn-RT and Mn@HFn-HT was calculated after measuring the relaxation time as a function of CAs concentration. Besides the r_1 values already discussed in the main text, this analysis revealed that both Mn@HFn-RT and Mn@HFn-HT are suitable as positive CAs with a r_2/r_1 rate of 2.3 (± 0.3) and 2.9 (± 0.4), respectively (Table 1).[3]

Afterwards, the dependence of the oxidation state of Mn from the reaction conditions was assessed by a colorimetric assay.[1] Briefly, leucoberbeline complexation with Mn^{3+}, Mn^{4+} or Mn^{7+} was detected at 620 nm by UV-vis spectroscopy analysis and oxidized Mn quantified by means of a calibration curve. The results (Figure 2b) indicated that in Mn@HFn-RT samples Mn retained the initial oxidation state (II), while after Mn@HFn-HT synthesis it was mainly oxidized (68 ± 14%).

In order to confirm the direct correlation of the lower r_1 detected using Mn@HFn-HT compared to Mn@HFn-RT with the extent of Mn ions oxidation, an aqueous solution of $MnCl_2$ was incubated at 65 °C for 7 h monitoring the solution relaxivity and the abundance of oxidized ions. The results revealed a gradual loss in relaxation power with time (Figure S2b) consistent with a progressive metal oxidation (Figure S2a).

Cell viability assay. As required prior to the in vivo experiments, the cellular toxicity of Mn@HFn nanocomplexes was investigated. For this purpose, a viability assay was conducted incubating TfR1-expressing cells HCC1954 (expression confirmed in Figure S3) with Mn@HFn-RT and Mn@HFn-

HT at the same protein concentration (0.1 mg/mL) and the cellular viability was evaluated at 24, 48 and 72 h by using MTT. In this experiment, unloaded HFn was used as a control to assess any nanocarrier-related effect, whereas $MnCl_2$ was tested at two different concentrations corresponding to the Mn ions amount contained in Mn@HFn-RT and Mn@HFn-HT samples (80 μM and 327 μM, respectively). The results showed in Figure S4 revealed that cell viability was slightly affected by Mn@HFn-RT (above 85% over time), while in cells incubated with Mn@HFn-HT the cellular toxicity was significantly higher exhibiting a cell viability around 50%. This effect could be attributable to higher metal concentration, which could alter the normal cell cycle. The latter hypothesis was confirmed comparing this data with the controls (HFn and $MnCl_2$): indeed, the protein itself does not impair the cellular growth, while free Mn^{2+} ions displayed a concentration-dependent effect on cell viability. These findings were confirmed also conducting the test with a different cell line (HeLa) with a significant overexpression of TfR1 (Figure S3 and S4).

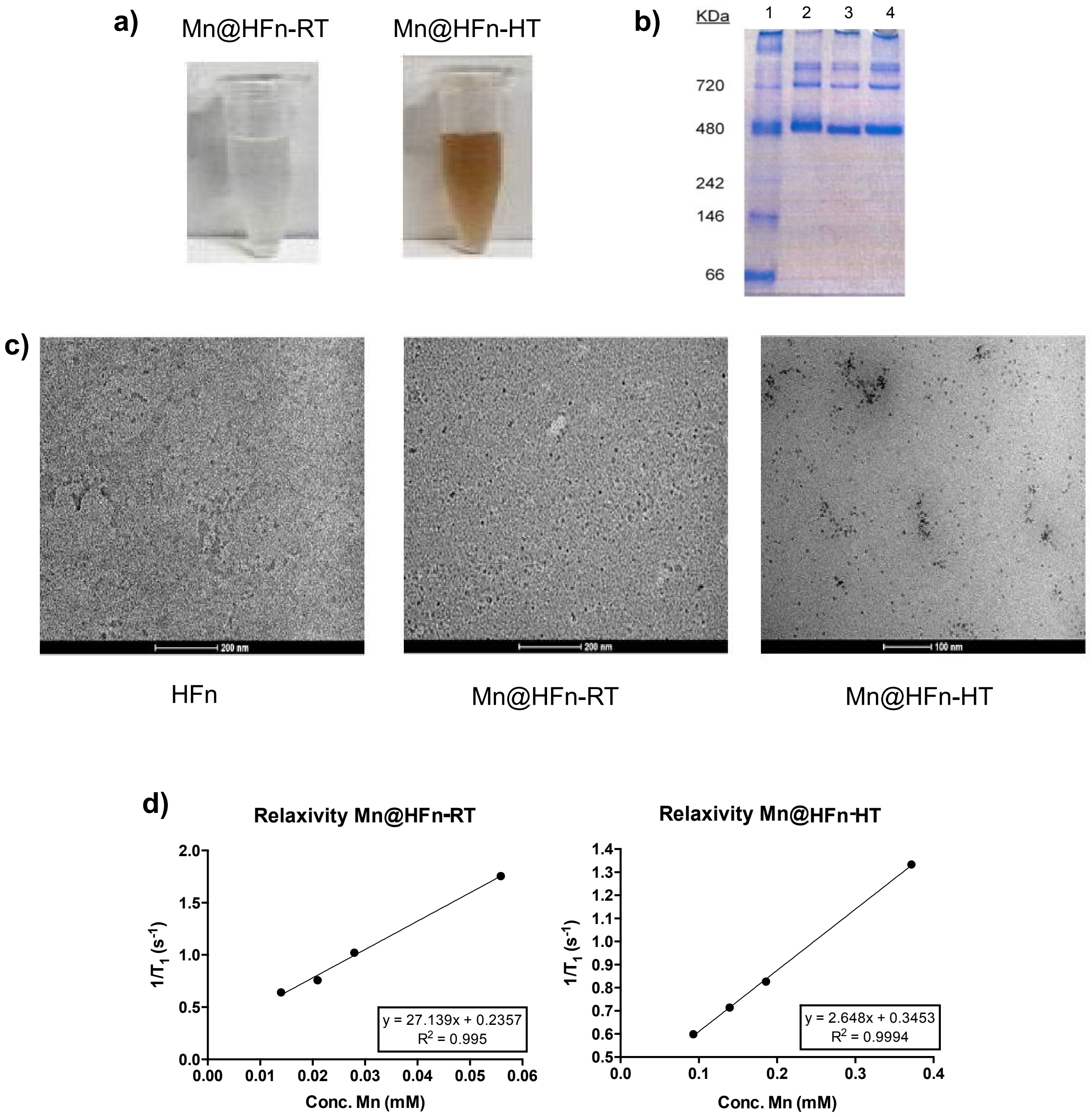

Figure S1 Nanocomplex characterization. a) Physical appearance of Mn@HFn-RT and Mn@HFn-HT. b) Native polyacrylamide gel 6% (*v/v*). Samples have been loaded in the following order: 1) marker, 2) HFn, 3) Mn@HFn-RT and 4) Mn@HFn-HT. c) TEM images of Hfn, Mn@HFn-RT and Mn@HFn-HT without uranyl acetate staining. d) T1 relaxation rate of representative batches of

Mn@HFn-RT and Mn@HFn-HT as a function of Mn molar concentration using 0.47 T NMR relaxometer. The slope of the curves expresses the transverse relaxivity (r_1).

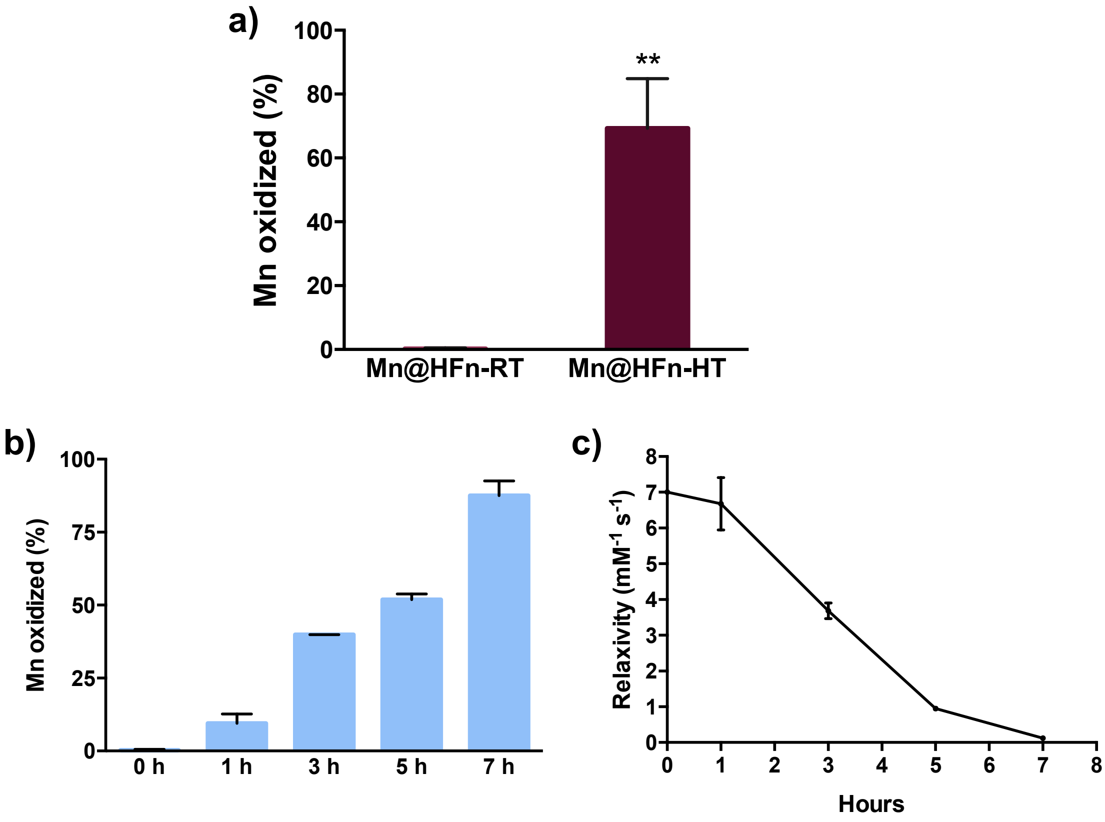

Figure S2 Mn oxidizes increasing the reaction temperature. a) Quantification of the percentage of Mn oxidized in Mn@HFn-RT and Mn@HFn-HT samples evaluated by leucoberbeline assay. Reported values are mean ± standard deviation (SD) (n = 3). b) Leucoberbeline assay performed on solutions of $MnCl_2$ left stirring at 65 °C. The graph shows the percentage of Mn oxidized as a function of time. Reported values are a mean of three replicates ± SD. c) Relaxivity values obtained measuring the relaxation time of a solution of $MnCl_2$ left stirring at 65 °C. The solution was checked every hour with a 0.47 T NMR relaxometer. Reported values are a mean of three replicates ± SD.

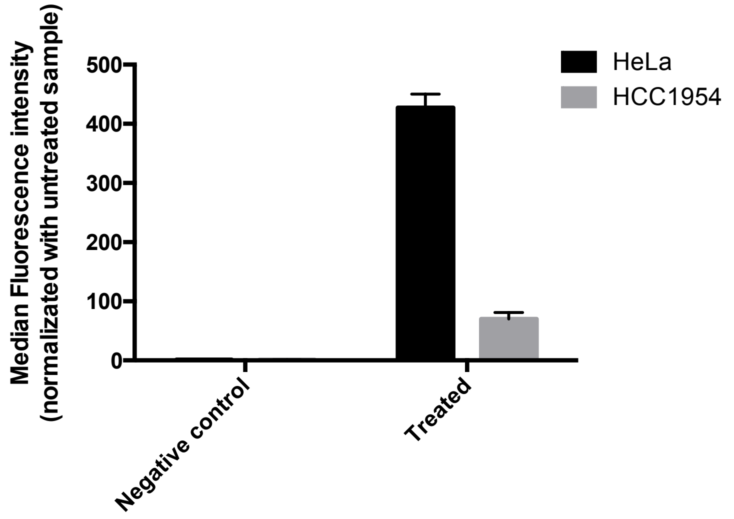

Figure S3. TfR1 expression in HeLa and HCC1954 cells evaluated by flow cytometry after double antibody immunostaining. Cells immunodecorated with the secondary antibody only were used as control. Reported values are the mean ± SD (n = 3).

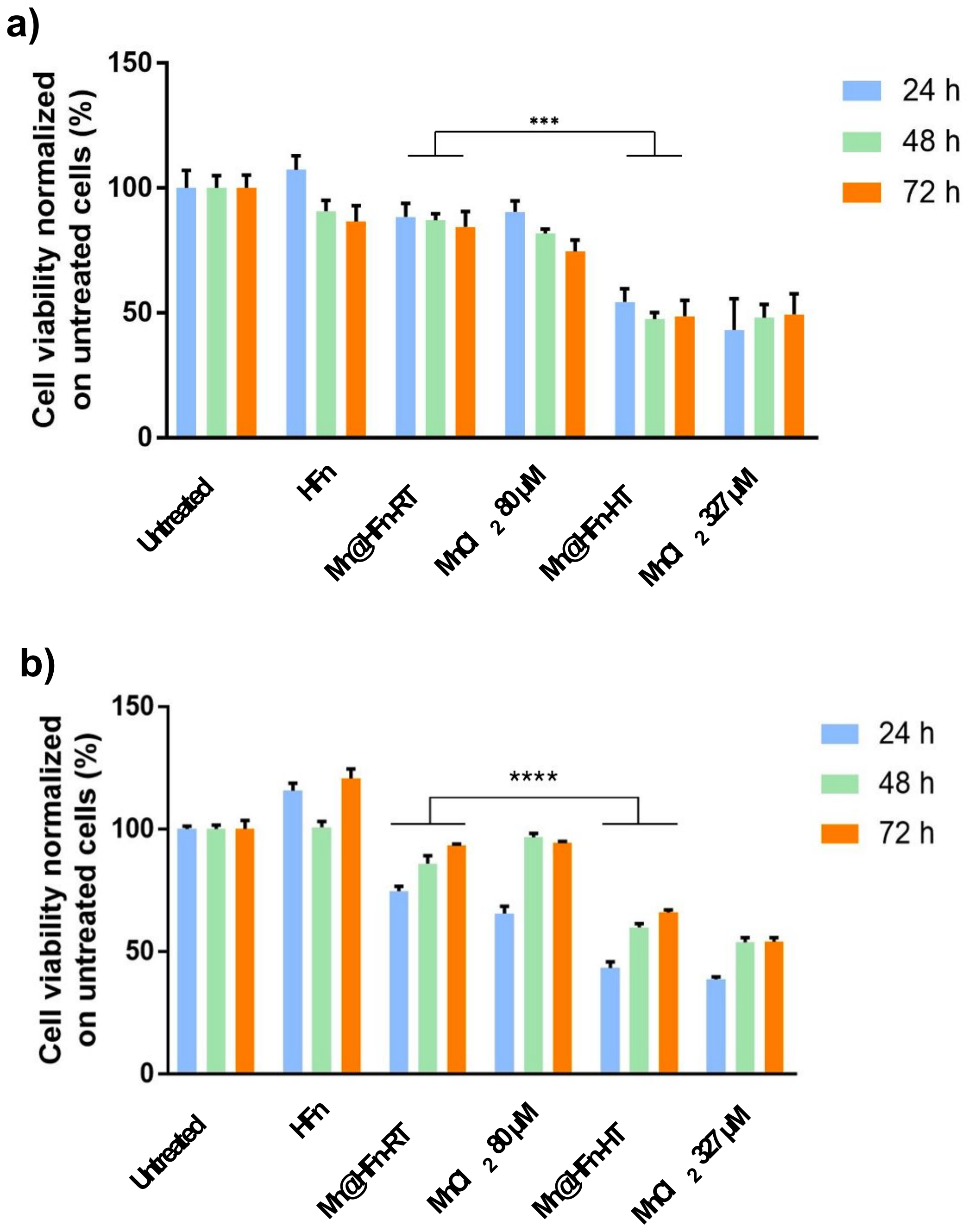

Figure S4. Cell Viability of TfR1-expressing cells (a) HCC1954 and b) HeLa treated with Mn@HFn-RT, Mn@HFn-HT, and the corresponding amount of HFn (0.1 mg/mL HFn) and $MnCl_2$ (80 µM and 327 µM $MnCl_2$). Reported values are the mean ± SD (n = 6) normalized on cell proliferation of untreated cells. *** $P < 0.005$ (Student's t-test).

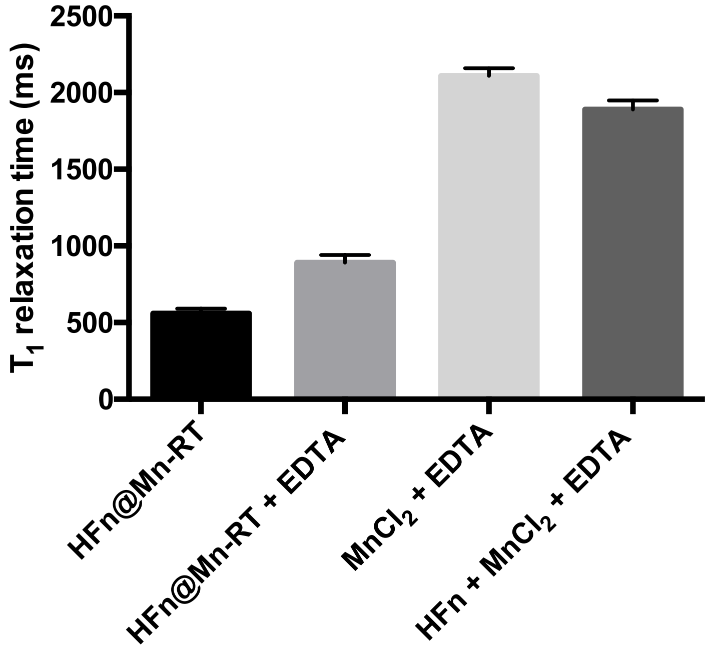

Figure S5. T_1 relaxation time measured with a 0.47 T NMR relaxometer of solution containing Mn@HFn-RT +/- EDTA, $MnCl_2$ + EDTA , co-incubating $MnCl_2$, HFn and EDTA. Notably, EDTA was added in 1:1 molar ratio with Mn. The values reported in the graph are a mean of three replicates ± SD.

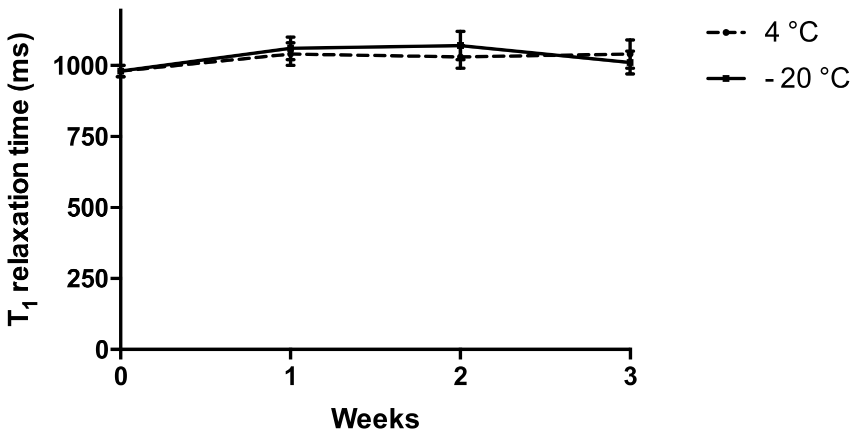

Figure S6. T_1 relaxation time measured with a 0.47 T NMR relaxometer on Mn@HFn-RT samples left for three weeks respectively at 4 °C and –20 °C. Each point is the average of four measurements.

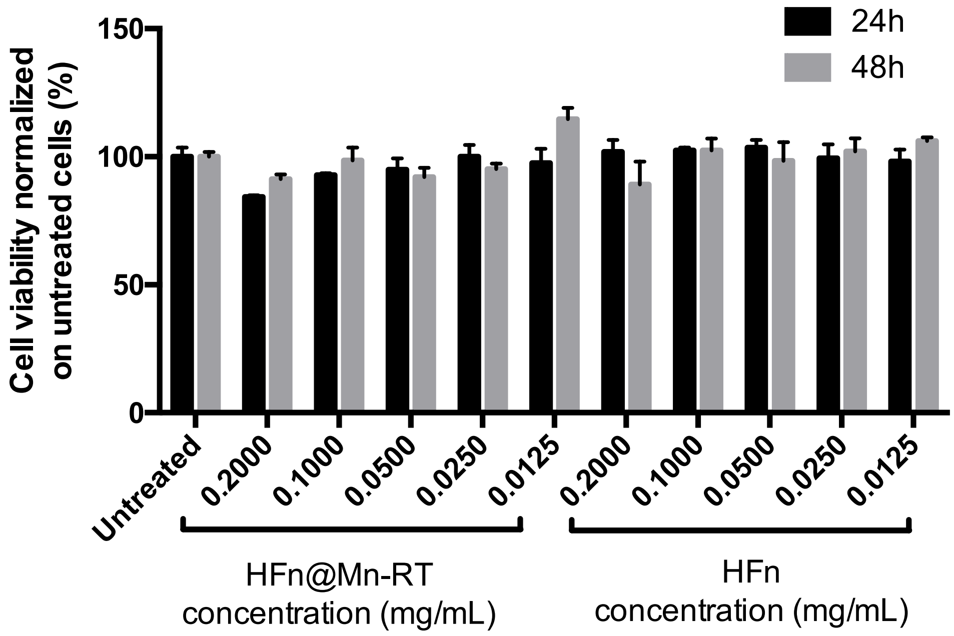

Figure S7. Cell Viability of HCC1954 treated with increasing concentration of Mn@HFn-RT and HFn. Reported values are the mean ± SD (n = 4) normalized on cell proliferation of untreated cells.

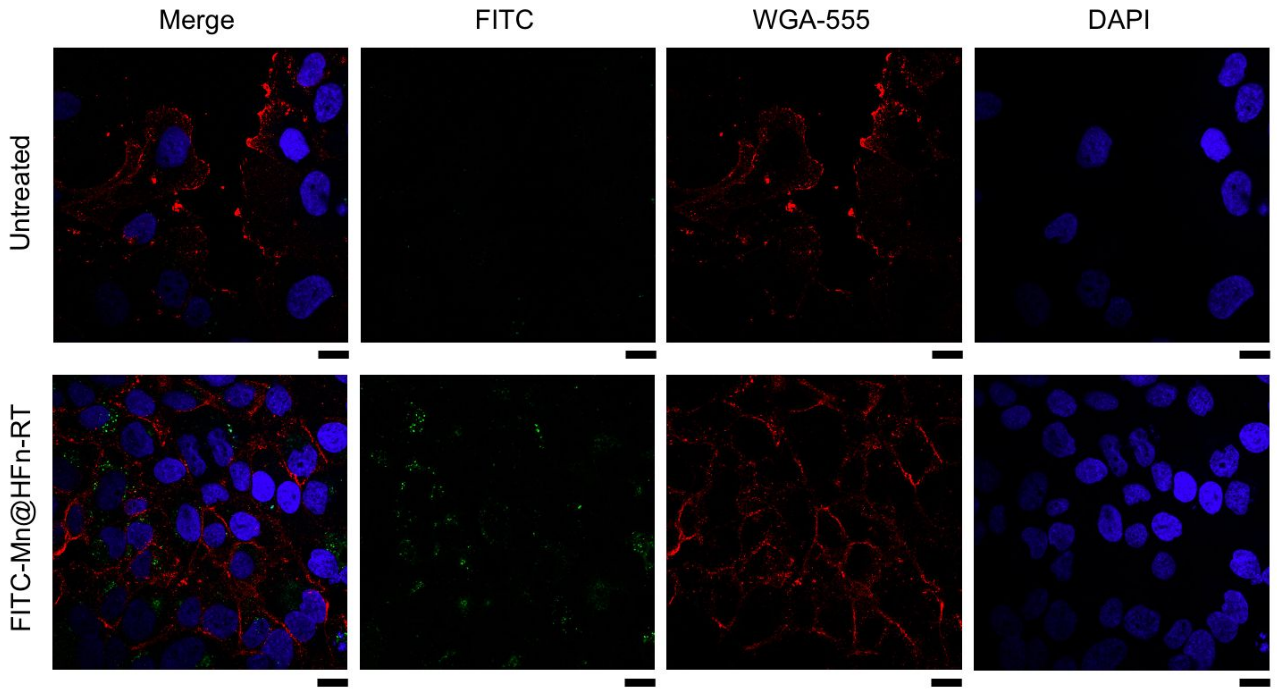

Figure S8. Confocal image of HCC1954 cells incubated (5 h, 37 °C) with FITC-Mn@HFn-RT (green; 0.1 mg/mL). Nuclei were stained with DAPI (blue), while the cell membrane with WGA labeled with 555 Alexa (red). The scale bar corresponds to 20 µm.

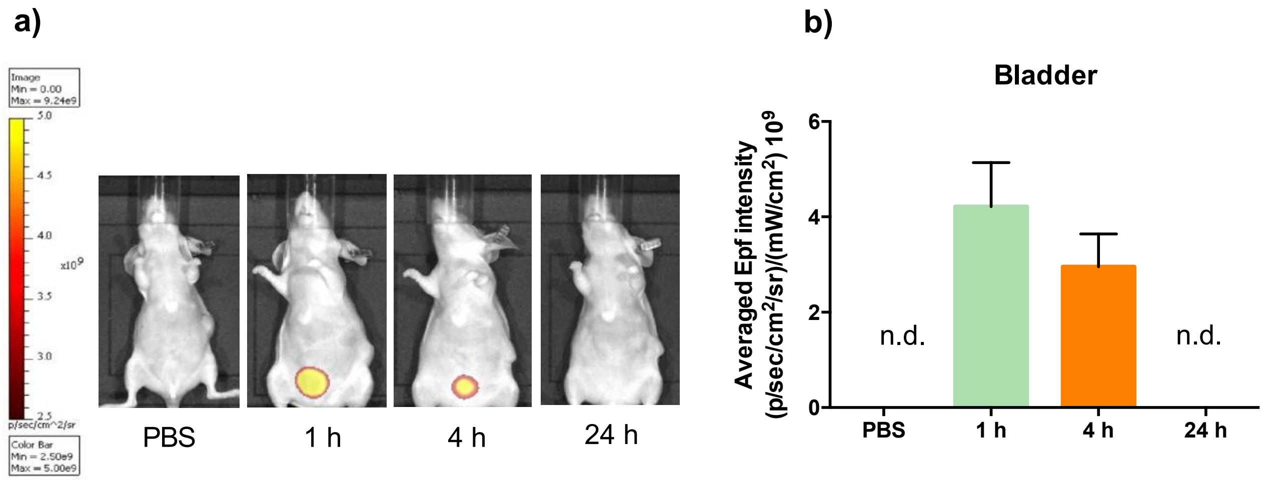

Figure S9. a) Epf images of representative mice HCC1954 tumors acquired 1 h, 4 h and 24 h after intravenous injection into the tail vein of 5 mg/kg of AF660-HFn. The color scale indicates the averaged epifluorescence expressed as radiant efficiency [(p/sec/cm^2/sr)/ (mW/cm^2)], where p/sec/cm^2/sr is the number of photons per second that leave a square centimeter of tissue and radiate into a solid angle of one steradian (sr). b) Averaged Epf intensity of the bladder region of interest (ROI). Reported values are mean ± SE (n = 3/4). PBS was administered as control.

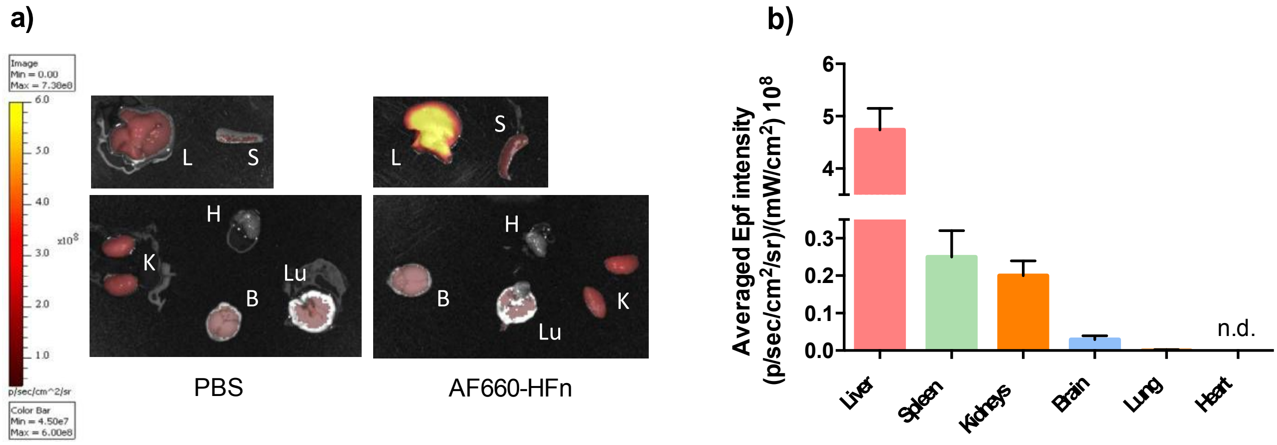

Figure S10. a) Epf of isolated spleen (S), kidneys (K), liver (L), brain (B), heart (H), lungs (Lu) after intravenous injection (4h) into the tail vein of PBS and 5 mg/kg of AF660-HFn respectively. The color scale indicates the averaged epifluorescence expressed as radiant efficiency [(p/sec/cm^2/sr)/ (mW/cm^2)], where p/sec/cm^2/sr is the number of photons per second that leave a square centimeter

of tissue and radiate into a solid angle of one steradian (sr). b) Averaged Epf intensity of the ROI obtained ex vivo after exposure to AF660-HFn. Reported values are mean ± SE (n = 3) normalized subtracting Epf values of PBS administered as control.

References:

1) Krumbein, W. E.; Altmann, H. J. A new method for the detection and enumeration of manganese oxidizing and reducing microorganisms. *Helgoländer Wiss. Meeresunters*. **1973**, *25*, 347–356. https://doi.org/10.1007/BF01611203

2) Ford, G. C.; Harrison, P. M.; Rice, D. W.; Smith, J. M. A.; Treffry, A.; White, J. L.; Yariv, J.; Miller, A.; Phillips, D. C.; Williams, R. J. P. Ferritin: design and formation of an iron-storage molecule. *Philos. Trans. R. Soc. Lond. B Biol. Sci.* **1984**, *304*, 551–565. https://doi.org/10.1098/rstb.1984.0046

3) Estelrich, J.; Sánchez-Martín, M. J.; Busquets, M.A. Nanoparticles in magnetic resonance imaging: from simple to dual contrast agents. *Int. J. Nanomedicine* **2015**, *10*, 1727–1741. http://doi.org/10.2147/IJN.S76501
